# Integrating Electronics to Textiles by Ultrasonic Welding for Cable-Driven Applications for Smart Textiles

**DOI:** 10.3390/ma14195735

**Published:** 2021-10-01

**Authors:** Sebastian Micus, Sahar Golmohammadi Rostami, Michael Haupt, Götz T. Gresser, Milad Alizadeh Meghrazi, Ladan Eskandarian

**Affiliations:** 1 German Institutes for Textile and Fiber Research Denkendorf (DITF), 73770 Denkendorf, Germany; michael.haupt@ditf.de (M.H.); goetz.gresser@ditf.de (G.T.G.); 2 Institute for Textile and Fiber Technologies (ITFT), University of Stuttgart, 70569 Stuttgart, Germany; 3 Research and Development Department, Myant Inc., Toronto, ON M9W 1B6, Canada; sahar.rostami@myant.ca (S.G.R.); miladam@myant.ca (M.A.M.); Ladan.eskandarian@myant.ca (L.E.)

**Keywords:** smart textiles, e-textiles, ultrasonic welding, electronics to textiles

## Abstract

The connection between flexible textiles and stiff electronic components has always been structurally weak and a limiting factor in the establishment of smart textiles in our everyday life. This paper focuses on the formation of reliable connections between conductive textiles and conventional litz wires using ultrasonic welding. The paper offers a promising approach to solving this problem. The electrical and mechanical performance of the samples were investigated after 15 and 30 wash-and-dry cycles in a laundry machine. Here the contact resistances and their peeling strength were measured. Furthermore, their connection properties were analysed in microsections. The resistance of the joints increased more than 300%, because the silver-coated wires suffered under the laundry cycles. Meanwhile, the mechanical strength during the peeling test decreased by only about 20% after 15 cycles and remained the same after 30 cycles. The good results obtained in this study suggest that ultrasonic welding offers a useful approach to the connection of textile electronics to conductive wires and to the manufacture of smart textiles.

## 1. Introduction

### 1.1. Smart Textiles and Level of Integration

Smart textiles are technical textiles with additional intelligent properties [1]. These novel products have been developed by many researchers for more than 20 years [2]. Smart textiles may interact with the environment by sensing external stimuli, adapting in a programmed way, and reacting accordingly. These specific functionalities and features are gaining in importance in the development of wearables and e-textiles. Intelligent and wearable textiles functionalized with electronics and electrical features are called e-textiles [1].

There will be a high growth potential for the smart textile market in the next ten years [3]. Electronics will soon be used in smart textiles to create hybrid products, to add new smart functionalities and to follow emerging trends in communication, customization, health, longevity, protection, performance, well-being, and the internet of things (IoTs). According to a report from IDTechEx [2], in 2025, over US$25 billion will be spent on formulations and intermediate materials for wearable technology. On the basis of the information in this report, the e-textiles market is predicted to reach US$3 billion by 2026. Sports and healthcare will be the two largest sectors in this field [2]. 

Smart textiles usually comprise sensors, actuators, data processing, a power supply, and a communication interface. The integration of smart textiles can refer to the incorporation of any conductive or electrical feature into any type of textile substrate. The conductive or electrical components of smart textiles are conductive yarns, conductors, semi-conductors, wires, printed circuit boards (PCBs) and conductive ink, which can be incorporated into conventional textiles. 

One of the main challenges for smart textiles is to maintain good performance once individual parts are integrated into the system [4,5]. Another important challenge is to increase their life cycle and reusability in order to overcome situations that create external exposure, such as the washing and drying processes [6,7]. Furthermore, combining dissimilar materials with different elasticities together to create a hybrid system with the desired functionality is another challenge in smart textiles development [8].

The attempts and efforts of many industries, professionals, and academic researchers to overcome integration challenges have helped to classify the integration techniques into three main categories [9,10]. 

The first-generation category of techniques is referred to as side-by-side or added-on technology, which includes any techniques that can incorporate, attach, or embed rigid electronic components, such as sewing, embroidery, and soldering, into the textile surface. Conventional sensors deliver much more precise data and provide a wide range of measurable values and opportunities. The main precaution when using wearables integrated with these techniques is to detach the electronics from the garment before washing or to protect the electronic components in a casing. Handwashing is also recommended for this type of product due to the fragility of its components, which do not have enough endurance in commercial or residential washing machines [9,11]. 

The extensive capabilities of textile machineries and manufacturing methods improved the level of integration that it was possible to achieve and helped to integrate electronics into fabric structures. This led to a more advanced form of integration, called built-in technology. By using various textile manufacturing methods, conductive components can be permanently integrated into textile structures to deliver a more hybrid system. These methods include printing and coating conductive ink on passive textiles, laminating printed electronics, knitting, weaving, and braiding the conductive yarn, fibre, and wire. Many industrial products on the market or under development may fit within this category. These products could be washed with commercial or industrial washing machines; however, their lifetime might be jeopardized after 25 wash cycles. Moreover, these types of electronics show a low level of conductivity, so they are not usable for many applications [9].

The development of conductive yarn manufacturing methods has led scholars to introduce the latest or third generation of integration level, which mainly aims to fully integrate and create smart materials with both textile and electrical functionality. Examples include creating an intrinsically conductive fibre with sensing and actuation capabilities, such as a transistor fibre, which is durable and washable. This is a challenging category and still requires a significant amount of research and development [9]. 

As of now, the smart textiles currently on the market are mainly a combination of conductive textile structures with rigid electronic parts [7]. Textile electrodes, strain and deformation sensors, capacitive sensors and heating applications are examples of the popular smart textile production chain [12]. Although conductive yarns can be used in fabric manufacturing processes and deliver a smart fabric, there is still a need for secured and light transmission lines, or a termination point, which cannot be always achieved with the conductive yarns. Conductive yarns can be metallic-based, such as carbon, or non-metallic based, such as metal fibre. These conductive yarns have been developed with conductivities ranging from five Ω/m to several kΩ/m [13]. Their low amount of conductivity makes conductive yarns fewer effective candidates for transmission lines in smart textile systems. Conductive yarns are generally not insulated, and they are not supposed to suffer from surface modification/oxidization either. However, it is hard to keep conductive yarns away from environmental exposures as they are embedded in substrates with tactile properties and in touch with water and moisture [13]. Therefore, insulation is crucial for conductive yarns if they are used as conductors for transferring signals. Besides, insulation increases the number of manufacturing steps for conductive yarns, which eventually affects their price. Therefore, the integration of available rigid conductors and semi-conductors into soft conductive textiles offers the most practical solution. Wires and PCBs are some of the main electrical components to be integrated into textiles [8,10]. There are cable-driven applications in smart textile products that use wires to make connections between sensors/electrodes/actuators to the terminal zone, such as the portable exoskeleton glove, which is used for object grasping and manipulation [14]. It is difficult to avoid exposing gloves to environmental features, such as water and moisture; therefore, the use of wire in multiple locations offers a safer option for transmission lines [14]. Other examples of cable-driven textile actuators are structured functional textiles in combination with a flexible actuation scheme that enables assistive torques to be applied to biological joints, such as soft exosuits for gait assistance [15] and shape memory alloy actuator-embedded smart clothes for ankle assistance [16]. In these devices, cables and wires are connected to textile-based components to provide mechanical linkage [9]. 

Advances in manufacturing have helped to develop the integration of flexible electronics into fabric structures. These have included fast-paced manufacturing methods, such as weaving narrow tapes with different gauges of insulated and non-insulated wires, the tailored wire placement (TWP) of wires on the fabric with e-embroidery and routing cables through garments during the knitting process on flat-bed machines. By embedding wires and conductors into textiles, the ease of attachment and aesthetics of a connection may be improved. However, physical strength, electrical reliability, durability, and low cost are yet to be achieved. There might be more than one location in smart textiles that requires a bond as a termination point between wires and other electronic devices. 

Scholars have identified nine main factors to consider when choosing a connection method between rigid electronics and textiles: physical strength, electrical reliability, ease of attachment, repeatable re-attachment, aesthetics, size, comfort, cost and availability. The main methods involved are: soldering, different types of welding, the use of conductive adhesive and the use of pressure-based connectors. Solder is an option for metal-based yarns that offers physical strength and electrical reliability; however, its higher processing temperature may burn or damage textiles and affect the durability and aesthetic aspects of a garment [17].

Electrically conductive adhesives have been introduced to provide mechanical strength and electrical reliability for the bonding method. This method has been compared to t solder technology and some disadvantages have been reported, such as lower electrical conductivity, conductivity fatigue in exposure to humidity, high temperature and wear and tear [17].

Another method to connect wires or electrical components firmly to textiles is to use pressure-based connectors. These connectors could be staples or crimping. Each method has an impact on the fabric’s properties. For example, staples can clamp the wires into place and offer a conductive pathway. The staples are considered non-reversible. Crimping uses some metal holders to penetrate the textile, in order to create an electrical connection between the connector and the conductive part. This method is non-reversible, and the connector must endure the wear and tear [17].

A quick bonding process that ensures physical strength and electrical reliability is required. As has already been stated, the existing method mainly involves several manual tasks and, in many cases, it requires commodity materials, such as conductive epoxy, solder paste, clips, and snaps. Furthermore, an extra encapsulation technique is required to secure the termination point from environmental exposure, including mechanical tensions and wash cycles [17]. 

### 1.2. Ultrasonic Welding as an Integration Technique for Smart Textiles

Ultrasonic welding is one of the most popular welding methods for joining and it has been used in numerous industrial fields, including the automotive, aerospace, medical, electronics and textiles industries [18]. This welding method uses mechanical vibrations to generate heat at the joint to soften and melt the materials in order to bond them. It is fast, economical, easily automated, and well-suited to mass production. As it does not introduce contaminants or sources of degradation to the weld that could possibly affect the biocompatibility of the medical device, it is a favorable joining method for medical applications [19]. It produces consistent, high-strength joints for seam sealing applications in the textile industry. Its quick weld time and elimination of the need for an extra ventilation system are other advantages of this welding method that make it suitable for mass production in the textile industry. Its other applications include strapping, belt loops, filters, and vertical blinds. 

The main applications of ultrasonic welding in the textile industry include cutting and seaming, slitting, trimming, embossing and quilting. These methods consume little energy, eliminate the need for commodity materials, such as glue, clips or needles and can be manufactured very quickly [20]. Ultrasonic vibrations and pressure cause heat-activated materials to melt and penetrate the inter-fibre spaces of fabrics. Many studies have been performed on different ultrasonic welding modes and their processing parameters, namely, weld time, weld pressure and hold time. Their results show that the ultrasonic welding of textile materials depends on their thermoplastic content, fabricating types (woven, knitted, non-woven) and desired end use [17]. The ultrasonic welding of thermoplastic substrates is divided into two categories, based on the position of the horn. In near-field welding, the distance between the joint interface and the horn is 6.35 mm or less, whereas in far-field welding, the distance is greater than 6.35 mm [21].

W. Shi [22] investigated the potential for building smart seams by incorporating optic fibres through ultrasonic welding machines. The morphologies of the optic fibres embedded were examined under varying welding conditions and the power loss in the signal transition of the incorporated optic fibres was tested to determine the degree of attenuation of their signal transition properties. The analysis of the power loss of the embedded optic fibres indicated that the signal transmission properties of the optic fibres were not significantly changed under certain welding conditions. Therefore, optic fibres can be integrated into fabrics ultrasonically [22]. 

O. Atalay [19] used ultrasonic welding to analyse its suitability for e-textile transmission line manufacturing. Two groups of silver-plated and stainless-steel yarn with different linear densities were used. The yarns were placed between two layers of polyester fabric and welded with ultrasonic seam-sealing machines. Based on the experimental results, the conductivity of the stainless-steel yarns barely changed; however, increasing the contact force resulted in the rupture of strands. Filament rupture was also observed in silver-plated nylon yarns after the contact point was increasing. The electrical resistance values showed significant fluctuations for the silver-plated nylon yarn, even at low contact force values. Therefore, stainless steel yarn can be considered a suitable candidate for textile transmission lines integrated with ultrasonic welding; however, the use of silver-plated nylon is not recommended. Welding the conductive yarns between layers of hydrophobic polyester fabric adds an encapsulation property to the transmission lines, which increases their durability by protecting them from water contact and probable short circuits [19]. 

J. Lesnikowski [18] studied the properties of textile signal lines (TSLs) made using ultrasonic a welding machine, model PFAFF 8310. Three plain-woven nickel metalized polyester fabrics with a length of 150 mm and a width of 35 mm were placed between two non-conductive layers of fabrics with different weaving constructions, including plain, satin and panama. In both constructions, two of the signal paths were used as ground paths. The linear resistances characterizing the properties of the TSLs working as direct current (DC) lines were measured. To check the signal integrity of the TSLs transmitting high-frequency signals, characteristic impedance and scattering parameters were described and measured. The bonding strength between the electroconductive path and passive fabric was measured using a tensile testing machine. The results of this research showed that despite the ability to produce TSLs capable of transmitting DC and alternating current (AC) signals offered by ultrasonic welding technology, the direct welding of electroconductive paths on fabric affected the TSLs’ functionality. Based on these observations, ultrasonic bonding has a destructive effect on electroconductive paths, and these effects cause partial or complete loss of electrical conductivity. The precise positioning of paths and the manual production process were described as difficulties involved in this integration technique. The increased stiffness properties of constructed TSLs compared with samples made with other technologies was described as another disadvantage of the ultrasonic welding method [18].

In this study, we went one step further. Textile conductive sensory surfaces have advantages over conventional electronics in terms of drapability and haptics. Textile sensors are already very well developed and researched and can already be manufactured. The conductivity of the yarns used in textile sensors cannot compete with that of metallic strands. However, the connection of textile sensor technology with conductive cables has been rather difficult up to now. Over longer distances, energy sources as well as electronic control units should be equipped with highly conductive cables. Since the connection of textile electronics and cables has not yet been investigated in detail, the use of this sensor technology is limited. Therefore, this article presents a method for bonding wires to the functional textiles with ultrasonic welding for cable-driven applications. 

## 2. Materials and Methods

**Figure 1:**
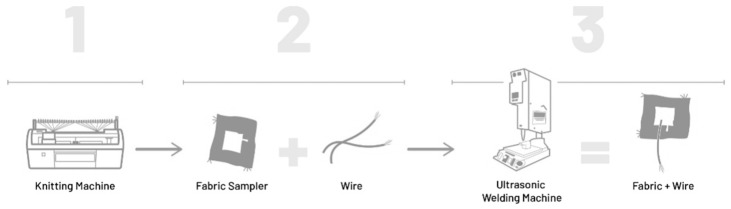
Schemes follow the same formatting.

In this study, we investigated a method for bonding wires to functional textiles with the ultrasonic welding method for cable-driven applications (Figure 1). This production method for smart textiles opens up totally new opportunities. The products of this combination of conventional electronics and textile sensors the products increase value for customers.

**Figure 2:**
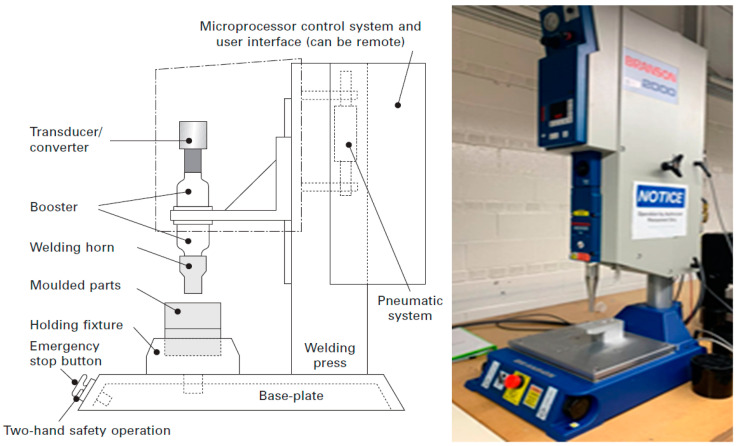
Schematic of the ultrasonic welding machine, displaying its key features [9] (**left**); Photograph of the ultrasonic welding machine (**right**).

During the assembly process, conductive knitted fabric was joined with a copper litz wire. In order to make the proper contact point between the silver yarns and the copper conductor, the conductor was stripped manually to about 10 mm and then inserted between the active textile and a waterproof tape to provide proper encapsulation. The samples were integrated using an ultrasonic welding machine (Emerson Electric Co.—Branson 2000; Saint Louis, MO, USA), as shown in Figure 2. The method involved a longer optimization process with an experimental design. During this process, there were several parameters involved that ensured the optimization of each joint between the two materials. In order to optimize the welding parameters for a bond, some visual characteristics were taken into consideration. As the method applies to to wearable technologies and its base materials are flexible textiles, one of the main factors involved in its visual characteristics is the avoidance of damage to the conductive fabric during the bonding process and of the possibility of burning and tearing due to overheating and thermal energy discharge from the horn during bonding. 

Over-melting wires and breaking the conductors are the other important factors to take into consideration. Over-melting the fabric around the wire might leave some residual chemicals on the fabric, which lead to imperfections on smart textiles and e-textiles. Besides, it is very important not to break the conductor strands under the anvil pressure as this might result in an unreliable joint for electrical applications, such as conductivity. For this reason, American Wire Gauge (AWG) 24 copper conductors were chosen, as they are soft and flexible. 

Finally, leaving a visible trace of the anvil shape behind the welded area would not be acceptable given the possibility that there might be more than one wire required for a garment. To avoid this effect, a rounded 10 mm-diameter threaded horn was chosen to weld the copper conductors to conductive fabric. Besides, the materials for the fabric were chosen based on their ultrasonic weldability. For instance, it was previously established that wool and cotton are not very suitable for ultrasonic welding due to their lack of thermoplastic content. The following parameters were used to bond the copper conductors to the conductive textiles with the ultrasonic welding machine (Table 1).

### 2.1. Sample Preparation in Knitting

**Figure 3:**
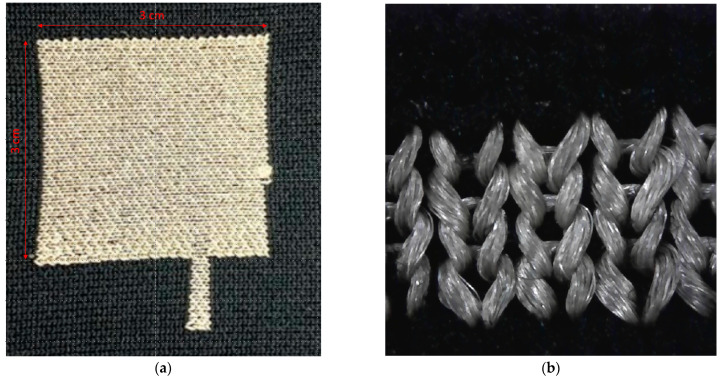
Picture of the knitted sensor (**a**) and knitting pattern (**b**).

The type of knit structure used is an important factor in ultrasonic bonding. The thickness of the fabric, whether there is elasticity in both directions and whether there is a tight or a loose textile structure all influence the bonding strength. For instance, an increase in fabric thickness would require an increase in weld time and amount of pressure, which may affect the bonding strength adversely. As our purpose is to use the bonding technique in smart textiles, the machine gauge was chosen based on the reproducibility of smart textiles on the knitting machines with finer gauges (Table 2). The active textile samples were knitted on an 18-gauge Stoll flat knitting machine. The silver-plated polyamide yarns were knitted on the surface of a double-knit structure in 3 cm by 3 cm (9 cm^2^) area. A small 2 mm by 8 mm tail was added to the square structure to make a junction for a further integration contact point. Polyester yarns were chosen for the passive parts of the knitted sample because of their suitability for ultrasonic welding. In order to provide the fabric’s structure with elasticity, one end of elastane was used as an interlocking connection between the two layers of knitted fabric (Figure 3) [17,21].

### 2.2. Test Scenarios

The electrical and mechanical properties of the joints in different test scenarios were investigated. The measurement of contact resistance is well suited to the characterisation of the electrical properties of connections. The contact resistance was measured within a four-wire resistance measurement setup. The mechanical properties of the contacts were examined in a peeling test. The test made it possible to determine whether the connection was robust enough for further production steps and as a product during wash and wear cycles. Furthermore, several microsections of the joints were taken from multiple replicates before and after washing.

#### 2.2.1. Electrical Properties: Contact Resistance Test

**Figure 4:**
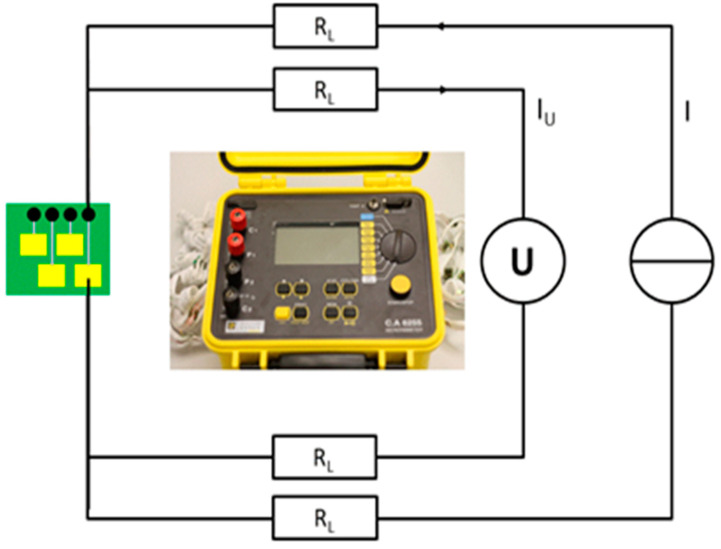
Measuring setup to determine the contact resistances using four-wire measuring technology.

Contact resistance is one of the decisive variables in the analysis of electrical contacts. The four-wire resistance test is particularly suitable for determining contact resistance because it makes it possible to measure only the contact resistance, excluding the line resistance of the measurement wires. When the contact resistance is not of a high value, the resistance of the cables becomes significant [23]. The contact resistance measurement can indicate the quality of the contact itself. A four-wire measuring instrument, in this case a micro-ohmmeter (Chauvin Arnoux Group—C.A 6255, Paris, France), was used to eliminate connection and line resistances in the test setup (Figure 4). 

In order to generate a measurable voltage, high currents of up to 10 A were required. The current was provided by two of the four contacts. The falling voltage at the resistor was measured by a voltmeter over the two remaining conductors. Based on Ohm’s law, it was possible to calculate the resistance of the contact. The contact resistances were in the range of only a few milliohms [24].

#### 2.2.2. Mechanical Properties: Peeling Test

A peeling test was carried out to determine the mechanical strength of the joint for further processing. It is a simple and fast method to analyse the adhesion of electronic interconnectors on ribbons [25]. Therefore, the self-developed peeling test is ideal for inspecting joints between PCBs and conductive textiles. 

**Figure 5:**
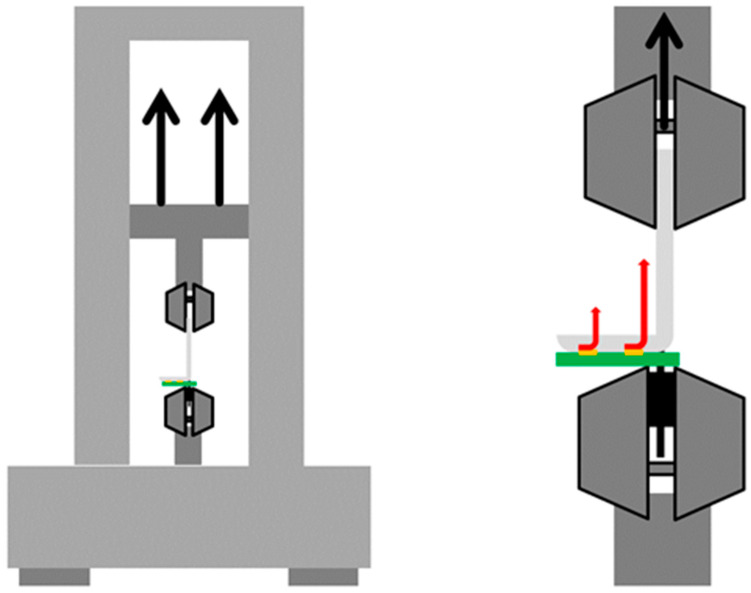
Peeling test setup, in which the samples were clamped to the blank and pulled on the attached wires with a speed of 100 mm/min.

The textiles were clamped on a table, which was fixed on the bottom section of the Zwick tensile testing machine (Z020, measuring head: 50 N). The conductors were peeled off the textile at a 90° angle. The force was applied perpendicularly to the textile (Figure 5). A load cell with a maximum force of 50 N was used at a travel speed of 100 mm/min [26,27,28]. 

## 3. Results and Discussion

### 3.1. Optical Properties: Microsection

**Figure 6:**
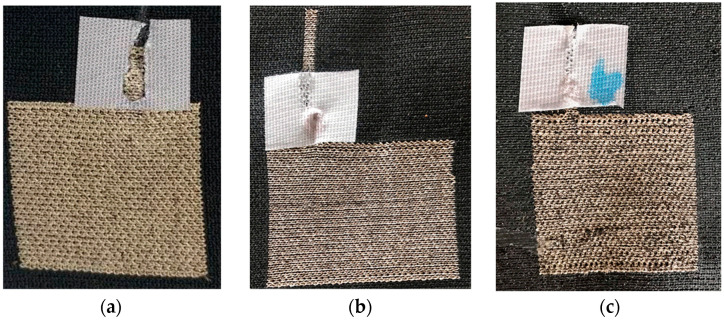
Photos from the samples: Unwashed (**a**), 15 timeswashed (**b**), 30 times washed (**c**).

Figure 6 shows pictures of the different samples: unwashed, 15× washed and 30× washed. The samples featured the same kitting parameters and the same tail structure, but the size of the textile electrodes differed. The size of the conductive electrode did not influence the connection process of the wire and the tail. The pictures showed structural alterations in the knitted fabric. The white waterproofed tape secured the connection and supported the connection area. 

In order to investigate the integrity of the bond between the copper conductors and the conductive fibers, SEM pictures were taken of the cross-section of the joint. Due to the pressure applied by the ultrasonic welding anvil during the bonding process, the copper conductors were completely adhered to the conductive fibers. 

**Figure 7:**
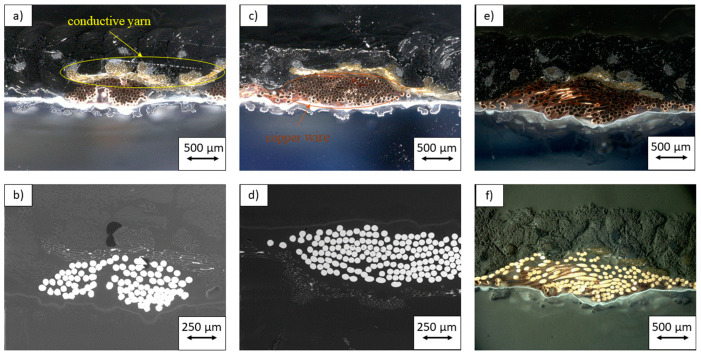
Microsections unwashed (**a**,**b**); 15× washed (**c**,**d**); 30× washed (**e**,**f**). Incident light microscope (upper row: **a**,**c**,**e**) and scanning electron microscope (SEM) (bottom row: **b**,**d**,**f**).

Figure 7 demonstrates microsections of the unwashed, 15 times washed and 30 times washed samples. The microsections were examined under an incident light microscope (KEYENCE—VHX-2000 Digitale Mikroskop, Osaka, Japan, upper row: a, c, e) and a scanning electron microscope (SEM: Hitachi—Tabletop Microscope TM-100, Chiyoda, Japan bottom row: b, d, f). The pictures illustrate the connection between the textile conductive yarns from the tail structure of the electrode and the metallic wire. Pictures (a) and (d) in particular show several contact points, which is a good sign for a low-resistance joint.

### 3.2. Electrical Properties: Contact Resistance Test

**Figure 8:**
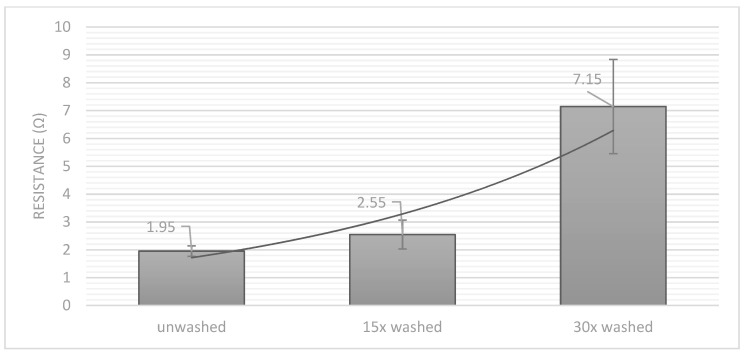
Contact resistance from the four-wire resistance test after different washing cycles (black exponential trend line).

In order to investigate the electrical properties and the quality of the connection, a four-wire resistance test was performed. As expected, the electrical resistance increased with the number of washing cycles. While the unwashed samples demonstrated a resistance of just 1.95 Ω, after 15 washing cycles the resistance reached 2.55 Ω and after 30 cycles the samples demonstrated a contact resistance of 7.15 Ω. Moreover, the standard deviation of the values rose with the number of washing cycles (Figure 8). The strands were connected by ultrasonic welding to silver-coated conductive yarns. The coating suffered heavily due to the mechanical and chemical stress during the washing process. In the beginning, there was enough coating on the yarn, so the first cycles did not have a high influence on the resistance of the connection. After repetitive wash and dry cycles, the coating was reduced in thickness and the resistance increased. In summary, it can be said that the number of washing cycles must be regulated to ensure the perfect functioning of smart textiles.

### 3.3. Mechanical Properties: Peeling Test

**Figure 9:**
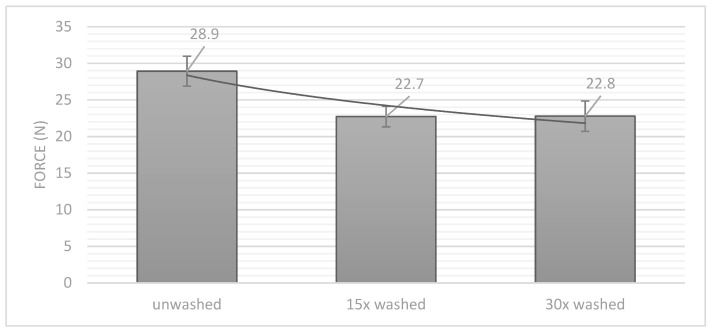
Peeling strength force after different washing cycles (black exponential trend line).

To analyse the mechanical properties of the connection, a peeling test was performed at aa 90° angle. The unwashed samples reached a strength of almost 29 N in the peeling test. After 15 times and 30 times cycles of washing, this value dropped to just under 23 N (Figure 9). When comparing the results with those of soldered joints [26], ultrasonic welding shows comparable values of 23 N. It can also be seen that the strength does not decrease further with the number of washing cycles, which indicates that the designed joints were very durable. The number of washing cycles did not have to be regulated due to the strength of the joints. Evidently, the connection was not influenced by mechanical stress during the washing cycle.

**Figure 10:**
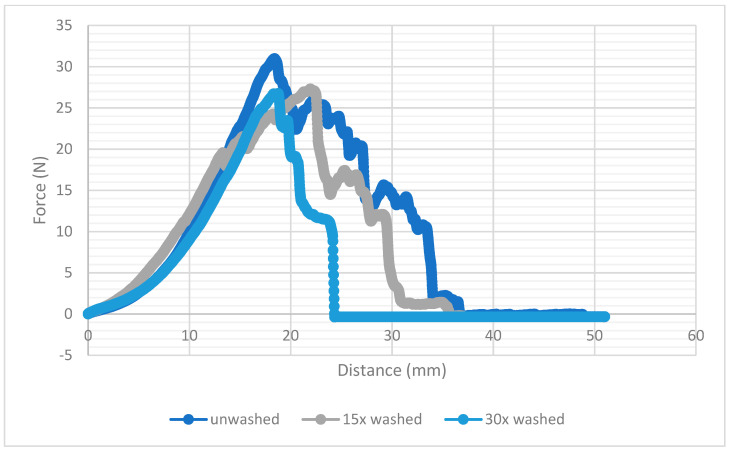
Peeling strength force after different washing cycles: course of force during the peeling.

Figure 10 illustrates the course of force during the peeling test for each sample. The graphs show an approximately uniform development. The washed samples demonstrated an irreducibly lower maximum force than the unwashed samples, but the course and the pitch angle were nearly the same. The results show the excellent mechanical properties of the joint. Both the unwashed and the washed samples demonstrated very high peel strengths. It can be concluded that the joints were hardly affected mechanically by the washing process. Figure 10 also shows that the maximum ductility of the joint decreased with the number of washing cycles. The entire structure thus became more brittle.

## 4. Conclusions

This paper dealt with the development of direct connections between conductive textile sensors and copper strands via ultrasonic welding. The application relevance and the advantages of wash-stable compounds was the reason for the investigation. The joining of conductive textile surfaces and wires helps to develop new smart textiles with the advantages of the usage of pure textile sensors and the high conductivity of metallic strands in clothes, for example. Textile sensors offer great opportunities for several smart textile applications in everyday life because the drapability and haptic quality of textiles make them suitable for wearing on the skin. The method replaces much larger snap fastener connections and enables the production of totally new smart textiles. For this purpose, we adapted the well-known ultrasonic welding technique to connect these joining partners. Therefore, we optimized the parameter-sets and produced the setup presented here. During the connection process, fabric and wire damage were considered. An increasing weld time and pressure would affect the bonding process adversely. The ultrasonic parameters were carefully examined and chosen in order to maintain proper bonding between the conductive fabric component and the wires. Afterwards, we examined the joining section of the samples, as a combination of wire and fabric damage. To ensure the usability of the resultant smart textile, we performed several washing tests. Some of the samples were washed 15 times and others 30 times. The samples were optically analysed under an incident light microscope and an SEM. Thus, microsections of the joints were made. The microsections clearly showed connections between the textile conductive yarns and the metallic strands. The electrical properties, especially the contact resistance, were investigated using a four-wire measurement setup to minimize the influence of the wire resistance. The resistance increased during the washing cycles because the silver coating of the conductive yarns suffered under the mechanical and chemical stress during the washing process. The coating washed away and rubbed off, so the conductivity decreased and the resistance increased exponentially. The mechanical properties of the connection were analysed during a peeling test, where the wire was peeled off at a 90° angle from the textile. At the time of writing, there is no equivalent method through which to measure the bonding strength of electrical components to textiles. As a result, the connections were hardly subject to any mechanical stress. The results of the washing cycles also proved that machine washing has no effect on bond strength. The presented method enables the secure and reliable connection of metallic wires to knitted textile sensors, as shown by the results of the washing tests and the peeling test. 

In summary, ultrasonic welding technology is an effective method for the connection of metallic wires to conductive textile sensors. The technology will significantly broaden the commercial applications of smart textiles.

## Figures and Tables

**Table 1:** Ultrasonic welding parameter.

Type of Horn (Head)	Weld Time (s)	Hold Time (s)	Pressure (Psi/Bar)	Trigger Force (N)	Amplitude (μm)	Near Field or Far Field
Threaded Horn with 8 mm diameter	0.070	1.5	20/1.37	15	50	Near Field

**Table 2:** Knitting parameter.

Conductive Yarn	Knitting Structure	Thickness (mm)	Stitch Density (Course × Wale)
Silver Plated Polyamide/Nylon 220 Denier	Double Jersey with Elastane interlock	1.8	4 courses × 10 wales = 40
